# Instrumented Timed Up and Go (iTUG): A Systematic Review of Parameters Across Healthy, Older, and Neurological Populations

**DOI:** 10.3390/jcm15093307

**Published:** 2026-04-26

**Authors:** Piotr Szaflik, Katarzyna Nowakowska-Lipiec

**Affiliations:** Department of Biomechatronics, Faculty of Biomedical Engineering, Silesian University of Technology, Roosevelta 40, 41-800 Zabrze, Poland; katarzyna.nowakowska-lipiec@polsl.pl

**Keywords:** instrumented Up and Go, test Up and Go, TUG, iTUG, Parkinson’s disease, multiple sclerosis, elderly

## Abstract

**Background**: The use of inertial measurement units (IMUs) in the Timed Up and Go (TUG) test enables the quantitative assessment of functional performance and mobility. It allows for the determination not only of the total test completion time, but also of the durations of individual phases, as well as the derivation of spatiotemporal gait parameters and turning velocity. The aim of this review article was to compile parameters of the instrumented Timed Up and Go (iTUG) test and to identify the parameters most commonly analyzed in populations of healthy adults, older adults, and patients with neurological disorders. **Methods**: A systematic literature search was conducted in the PubMed, Scopus, and ScienceDirect databases. The authors included studies in which commercial IMUs were used during the TUG test and quantitative parameters were analyzed. Methodological quality was assessed using the JBI Critical Appraisal Checklist for cross-sectional studies. **Results**: A total of 36 studies were included in the review. Only those disease entities represented by at least four studies were included in the tabular analysis. The study presents results for a total of 1268 individuals, including 192 healthy adults, 514 older adults, 230 patients with multiple sclerosis (MS), and 332 patients with Parkinson’s disease (PD). The analysis showed that temporal parameters, particularly the total test duration and the durations of individual phases, were the most commonly reported across all populations. **Conclusions**: Turning-related parameters were analyzed frequently, whereas spatiotemporal parameters were assessed less often. The results indicate a lack of standardization both in the selection of iTUG parameters as well as in the measurement methods and systems used.

## 1. Introduction

Recent years have seen a rapid development of digital technologies supporting the assessment of motor function in medicine and rehabilitation [1,2]. The use of digital tools in diagnostics enables a more objective and precise assessment of movement parameters and their changes, supporting the work of physicians, physical therapists, and exercise professionals [3]. The use of motion capture systems, including inertial measurement units (IMUs) [1,4], marker-based systems [5] and markerless systems [6], enables the acquisition of detailed movement parameters during the performance of functional tests used in clinical practice for many years [7].

An example of such a test is the Timed Up and Go (TUG) test, which was developed in 1991 by Podsiadlo and Richardson [8]. The test is considered a basic assessment of mobility. It is widely used both in older adults and in patients with neurodegenerative diseases. In older adults, the TUG test is used as a screening tool to assess fall risk [9,10], whereas in patients with neurological disorders, it may provide information on impairments in balance and motor coordination [11,12]. Furthermore, the test is used not only in diagnostics but also in monitoring treatment outcomes [13,14]. The test begins with the participant seated on a chair. The participant then stands up, walks a distance of three meters, turns around, and returns the same distance to the chair, where they sit down again [8]. In the conventional assessment, physical therapists measure only the total test duration using a stopwatch. This does not allow for the analysis of velocity, movement strategies, or the identification of phases in which difficulties occur.

Digital technologies make it possible for the Timed Up and Go test to be equipped with inertial measurement units (IMUs). When performed using such sensors, the test is referred to in the literature as the instrumented Timed Up and Go (iTUG). Inertial measurement units (IMUs) enable the recording of movement parameters such as linear accelerations, angular velocities, and body segment orientation. Based on the recorded signals, it is possible not only to determine the total test duration, which has traditionally been assessed by a physical therapist, but also to automatically identify individual test phases, such as the sit-to-stand, walking, and turning phases. The analysis of these data enables the derivation of additional kinematic parameters, including movement velocity and acceleration during the performance of individual test phases. Additionally, the walking phase in the TUG test, performed over a distance of approximately 3 m, allows for a basic analysis of gait parameters [15].

The iTUG is increasingly used to assess functional performance and mobility. Various clinical populations are studied [10,16,17,18], and different inertial sensor–based systems are used for data acquisition. Some researchers use custom IMUs or self-developed algorithms to detect phases of the Timed Up and Go test [19,20]. As a result, numerous parameters describing test performance are reported in the literature, including, among others, the durations of individual phases, turning velocities, and kinematic and spatiotemporal parameters. However, a significant issue that remains is the considerable heterogeneity in the selection of parameters, analysis methods, and measurement systems used, which makes it difficult to compare results across studies. The lack of standardization hinders the interpretation of results and limits the implementation of the iTUG in routine clinical practice. Despite the growing number of studies, there is a lack of a comprehensive synthesis of reported parameters and their usefulness in relation to age and disease entity.

The aim of this review article was to compile a set of iTUG test parameters and to identify the parameters most commonly analyzed in studies involving different study populations.

## 2. Materials and Methods

### 2.1. Search Strategy

Between January 2026 and February 2026, a systematic search of the ScienceDirect, PubMed, and Scopus databases was conducted to identify studies using the instrumented Timed Up and Go test. The searches were performed on 5 February 2026 (PubMed), 3 February 2026 (Scopus), and 5 February 2026 (ScienceDirect). The search was limited to studies published between 2010 and 2026. This systematic review was conducted in accordance with the PRISMA guidelines [21] and it is included as Supplementary Files.

No additional filters were applied in any database. All searches were restricted to title and abstract fields where database functionality allowed.

The following search strategy was used in the PubMed database:

(“instrumented timed up and go” [Title/Abstract] OR “instrumented TUG” [Title/Abstract] OR iTUG [Title/Abstract] OR “sensor-based TUG” [Title/Abstract] OR “wearable-based TUG” [Title/Abstract] OR (“timed up and go” [Title/Abstract] AND (“inertial sensor” [Title/Abstract] OR IMU [Title/Abstract] OR accelerometer [Title/Abstract] OR gyroscope [Title/Abstract] OR “wearable sensor” [Title/Abstract] OR “body-worn sensor” [Title/Abstract]))).

In Scopus, the search strategy was adapted to the database syntax using TITLE-ABS-KEY fields. As Scopus does not use controlled vocabulary such as MeSH, the search was based on keyword combinations equivalent to those used in PubMed. The following query was applied: (TITLE-ABS-KEY (“instrumented timed up and go” OR “instrumented TUG” OR iTUG OR “sensor-based TUG” OR “wearable-based TUG” OR (“timed up and go” AND (“inertial sensor” OR IMU OR accelerometer OR gyroscope OR “wearable sensor” OR “body-worn sensor”)))). In ScienceDirect, the search was conducted using keyword-based queries applied to titles, abstracts, and keywords, in accordance with the platform’s search functionality. Due to the limited availability of advanced field-specific query options compared to PubMed and Scopus, a simplified search strategy was employed, based on the same combination of keywords as used in Scopus.

Differences in query syntax across databases reflect their distinct indexing structures and search functionalities.

**Figure 1:**
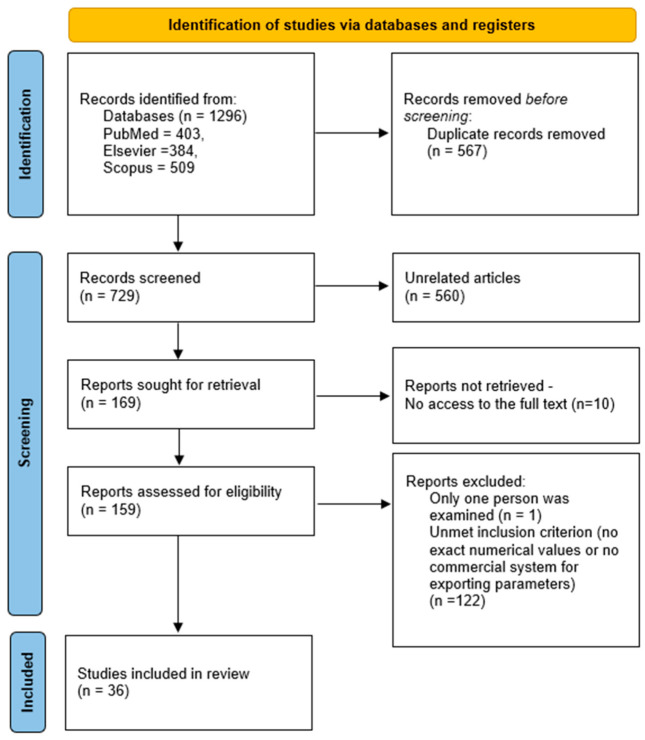
PRISMA flow diagram for the systematic review.

The search results were exported from the databases and imported into Rayyan, where they were merged. Duplicates were initially identified using Rayyan’s built-in algorithm, which relies on bibliographic metadata (including title, authors, publication year, journal, and DOI). Additionally, records with a high degree of similarity (threshold > 0.90) were flagged to identify potential duplicates that may not have been detected automatically. All identified records were then manually verified by the researchers, and the decision to remove a duplicate was made on a case-by-case basis following individual assessment. This approach was intended to minimize the risk of incorrectly excluding relevant publications. In the next stage, the titles and abstracts were screened to identify potentially eligible publications. Screening was conducted independently by two reviewers, including a preliminary assessment of eligibility criteria, such as the availability of numerical data and the use of commercially available systems equipped with IMUs for detecting TUG test phases. Figure 1 presents subsequent steps in the search for articles, along with the number of articles which comply with the assumed criteria.

### 2.2. Elegibility Criteria

The studies were selected using the PICOS criteria. The inclusion and exclusion criteria are presented in Table 1. The authors included only cross-sectional studies in which the TUG test was performed using commercially available IMU-based systems enabling the automatic detection of individual test phases and the derivation of their parameters. Additionally, the criterion was restricted to articles reporting parameter values as means and standard deviations, which allowed for comparison and tabular presentation of the results. Two reviewers independently extracted the data, and any discrepancies were identified and resolved through discussion.

### 2.3. Quality Assessment

All studies included in this systematic review were cross-sectional in design. Therefore, the Joanna Briggs Institute (JBI) Critical Appraisal Checklist for Analytical Cross-Sectional Studies was selected as the most appropriate tool for methodological quality assessment [22]. The assessment was performed by two independent reviewers. The checklist comprised eight questions and assessed whether the article clearly defined the inclusion criteria, the quality of the population description, the reliability and standardization of measurements, and the analysis of results. Each item of the JBI checklist was rated as “Yes,” “No,” or “Unclear.” For descriptive purposes, studies meeting 7–8 criteria were considered to have a low risk of bias, those meeting 4–6 criteria a moderate risk, and those meeting ≤3 criteria a high risk of bias. Disagreements between reviewers were resolved through discussion and consensus.

### 2.4. Risk of Bias Across Studies

Risk of bias across studies, including potential publication bias, was assessed qualitatively. Due to substantial clinical and methodological heterogeneity, a formal quantitative assessment (e.g., funnel plot or Egger’s test) was not performed. The assessment considered factors such as selective reporting and the potential overrepresentation of statistically significant findings.

### 2.5. Data Synthesis/Analysis

Due to the heterogeneity of the analyzed clinical populations and the limited number of studies addressing certain disease entities, a comparative synthesis of reported iTUG parameters was performed only for disease groups represented by at least four independent studies. Conditions represented by a smaller number of publications were described narratively, without conducting a detailed comparative analysis.

To focus on the most frequently analyzed parameters, only parameters reported in at least four different articles were selected for analysis. Additionally, articles that did not report the parameters included in a given table were not included in that table.

Due to substantial heterogeneity in study protocols, sensor configurations, and reported outcome measures, a quantitative meta-analysis was not feasible. Therefore, a qualitative synthesis of the results was performed.

## 3. Results

Following the literature review, 36 articles meeting the predefined PICOS criteria were included in the analysis. During the selection stage, 123 articles that did not meet the predefined inclusion criteria were excluded. The reasons for exclusion included studies conducted in children populations, the lack of analysis of quantitative iTUG parameters, and the use of nonstandard or noncommercial measurement systems. The detailed study selection process is presented in the PRISMA flow diagram (Figure 1).

### 3.1. Methodological Quality Results

The methodological quality of the included studies was assessed using the JBI Checklist for Analytical Cross-Sectional Studies. The results of the assessment are presented in Table 2.

Among the 36 included articles, 2 were classified as having a low risk of bias, while 34 were classified as having a moderate risk of bias. None of the included studies were classified as having a high risk of bias.

Due to the criterion of at least four studies per disease entity, which enabled the determination of the range of reported parameter values, 21 articles were included in the subsequent comparative analysis.

In two of the 21 studies, the analysis also included other clinical populations that did not meet the criterion of at least four studies. They were presented in the tables; however, only the results for Parkinson’s disease (PD) were included in the comparative analysis, while the remaining ones were presented in the Other Populations section.

The remaining 15 articles, concerning populations represented by fewer than four studies, were described narratively in the section Other Clinical Populations (<4 studies).

The majority of included studies were classified as having a moderate risk of bias, primarily due to insufficient reporting of confounding factors and limited information on measurement reliability. Only a small number of studies fulfilled all quality criteria, indicating an overall moderate methodological quality of the available evidence.

### 3.2. Characteristics of Included Studies

The included studies analyzed various patient groups. Table 3 includes only those articles concerning study groups represented in at least four studies, which allowed for the presentation of the range of reported parameter values. Based on the conducted analysis, the most frequently analyzed populations in the selected group of articles were healthy adults [23,24,25,26,27], older adults [9,27,28,29,30,31], patients with multiple sclerosis (MS) [16,32,33,34,35] and patients with Parkinson’s disease (PD) [13,18,36,37,38].

The walking phase was most commonly performed over a distance of 3 m [9,16,23,26,28,29,30,31,32,33,35,36,37], although distances of 6 m [18], 3.5 m [24] and 7 m [13,25,34,38] were also used.

In many articles, systems such as G-Walk [11,26,27,28,29,30,31,32,33,37], Mobility Lab [9,13,18,24,25,34,35,36,38], Noraxon [16,23] are used to segment the TUG test into periods (phases). Detailed information on the study groups, the systems used, and their placement is presented in Table 3.

### 3.3. Duration of the Entire Test and TUG Test Phases

In the analyzed articles, the total iTUG duration was most commonly examined. However, when considering the total duration or the duration of the walking phase, the measurement methodology must be taken into account, that is, how the participants were instructed. In most cases, participants moved at a natural, comfortable pace; however, some studies instructed participants to “walk as fast as”—TUG fast [28]. An additional factor that may affect the obtained temporal values is variation in the test protocol, particularly the length of the walking distance used in the TUG test.

In addition to the total test duration, some authors also analyzed the durations of individual iTUG phases, such as the sit-to-stand, walking, and turning. It should be noted, however, that not all authors reported the same test phases, which makes direct comparison of results across studies difficult. The reported values are presented in Table 4.

### 3.4. Gait Parameters

Table 5 presents the reported spatiotemporal parameters obtained during the walking phase of the iTUG. The omitted articles did not analyze the turning phases. The table also presents parameters that were most frequently reported by the authors.

Gait during the iTUG test was analyzed in 9 articles. The most frequently reported parameter was cadence, followed by double support, stance time, swing time, walking speed, stride length, and walking cycle time.

### 3.5. Turning Parameters

The turning phase is one of the more demanding components of the iTUG test, as it requires simultaneous control, coordination, and a change in direction. Table 6 presents the most frequently reported parameters of the turning phase in the iTUG test. The articles omitted from the table did not analyze the turning phases.

Overall, the turning phases were analyzed in 13 articles. The most frequently reported parameters were the maximum velocity of the second turning, followed by the mean velocity of the second turning, the mean and maximum velocity of turning 1, and the mean velocity of turnings 1 and 2.

### 3.6. Other Clinical Populations (<4 Studies)

Several additional clinical populations were identified in the reviewed studies; however, they were represented by fewer than four publications. Due to the limited number of studies, these conditions were not included in the comparative synthesis of parameter ranges and are instead presented below in a qualitative description.

The aim of Buraschi et al. [49] was to analyze temporal and kinematic parameters in the iTUG. The authors conducted a study on a group of older adults with a mean age of approximately 61 years with low back pain (LBP), along with an age-matched healthy control group. They found significant differences in the following parameters: total TUG duration, sit-to-stand duration, forward walking phase, return walking phase, final turning and stand-to-sit phase, mean velocity of turning 1, and accelerations during sit-to-stand and stand-to-sit phases. The authors concluded that IMUs are a valuable tool for assessing parameters derived from the TUG test and that their use in LBP provides valuable information enabling objective analysis. In turn, the aim of Celletti et al. [14] was to assess therapy for the treatment of the lower spine in older adults. Participants underwent the iTUG test before and after therapy consisting of 10 h of sessions scheduled three times per week, during which they performed various muscle-strengthening exercises. Using this test, they concluded that the training program is a promising new rehabilitative treatment for LBP in improving motor function and reducing functional limitations. The results obtained by the authors indicate significant pre- to post-intervention differences in parameters such as total test duration, sit-to-stand duration, and turning velocity.

There are also studies that investigate differences in iTUG performance among patients after stroke. For example, Spina et al. [41] reported significant differences in iTUG phases and turning velocities between patients after stroke and healthy control participants, suggesting that turning velocity during the Timed Up and Go test may be a sensitive indicator of mobility impairment after stroke. In another study, Tajuddin et al. [45] examined different task conditions (single task, dual motor task, and dual cognitive task) and found that dual-task conditions had a significant effect on gait parameters and increased total test duration in the iTUG, particularly during the dual cognitive task.

The literature includes a study by Winiarski et al. [42] that aimed to determine the long-term effects of COVID-19 on gait and the Timed Up and Go test. The study was conducted in a group of individuals who had been hospitalized due to COVID-19 and for whom more than 8 weeks had elapsed since discharge. The authors reported significant differences in the following parameters: total TUG duration, sit-to-stand duration, walking cycle time, mean speed, mean walking speed, and walking cadence, and observed differences in range of motion compared with individuals who had not been infected with COVID-19.

Assessments using iTUG were conducted by Cimolin et al. [44] in individuals with obesity, with a mean BMI of 41.1 ± 7.9 kg/m^2^. The authors reported differences in parameters such as total TUG duration, walking phase duration, turning 1 and 2 durations, and turning velocities. The obese group also exhibited increased maximum extension. At the same time, the authors concluded that iTUG is an objective and rapid mobility test and confirmed that it provides valuable information in clinical practice.

Arippa et al. [46] conducted iTUG assessments in individuals who had experienced severe traumatic brain injury. The aim of this study was to quantitatively assess the extent of motor deficits under both static and dynamic conditions. In assessing postural stability, the authors found a significant difference in sway area, and in the TUG test, in total walking time. They concluded that the measurements constitute a reliable tool and that their integration with clinical data is highly important. Prasad et al. [53] conducted similar studies in individuals after traumatic brain injury. The results showed that participants had reduced ankle activity compared with healthy individuals.

Familiari et al. [48] conducted a study to determine whether a shoulder abduction brace after arthroscopic rotator cuff repair affects gait parameters and functional mobility. The authors demonstrated that the brace affects walking speed after surgery, whereas no long-term effect was observed (one week after brace removal).

Monaghan et al. [43] and Mostile et al. [50] conducted iTUG studies in groups with essential tremor (ET). Based on both studies, it can be concluded that the iTUG test may provide valuable information.

Studies have also been conducted in individuals with facioscapulohumeral muscular dystrophy (FSHD). Statland et al. [52] reported that iTUG duration remained unchanged, whereas step length, step velocity, and trunk range of motion changed. Huisinga et al. [39] described motion capture systems as an appealing option for FSHD clinical trials.

Celletti et al. conducted a study in individuals with cervical dystonia [40]. The results were characterized by a significantly longer duration in patients with cervical dystonia compared with the control group, whereas no differences in flexion and extension angular amplitudes were observed. The findings indicate impairment in postural control during walking and postural changes.

In two studies, patients with fragile X–associated tremor/ataxia syndrome (FXTAS) were examined, where one study also analyzed patients with Parkinson’s disease (PD). Due to the limited number of studies, results for FXTAS were not included in the comparative synthesis of parameter ranges. Robertson-Dick et al. [38] conducted a study in groups with FXTAS, Parkinson’s disease (PD), and essential tremor (ET). Based on the results, the authors concluded that patients with FXTAS and ET exhibit different walking profiles than patients with PD. In turn, O’Keefe [47] conducted a study in patients with FXTAS with and without the premutation of the fragile X mental retardation 1 (FMR1) gene. PM carriers with FXTAS were globally impaired across all gait performance domains. Carriers without FXTAS did not differ from controls in any parameters, although double-limb support time was close to significance.

The literature includes a study in which patients with idiopathic normal pressure hydrocephalus (iNPH) and patients with Parkinson’s disease (PD) were examined (results for PD patients are presented in earlier tables) [37]. The authors of the study showed that patients with iNPH-P exhibited specific impairments in motor performance compared with untreated patients with PD, particularly during the transition from the turning phase to the stand-to-sit phase.

## 4. Discussion

The instrumented Timed Up and Go (iTUG) test is increasingly used as an objective tool for the assessment of mobility, walking, and balance in various clinical populations [16,29,35,36]. The aim of this review was to compile a set of iTUG parameters and to identify the parameters most commonly analyzed in studies involving different study populations. The analysis revealed differences between studies that included not only the measurement systems used, but also variations in TUG protocol methodology and participants at different stages of disease.

The selected articles were assessed using the JBI Checklist for Analytical Cross-Sectional Studies. Only 5.56% of the articles were rated as low risk of bias, whereas 94.44% were classified as moderate risk of bias.

A detailed analysis was conducted for articles concerning clinical populations represented at least four times. In total, the analyzed studies included 1268 participants: 192 healthy adults [23,24,25,26,27], 514 older adults [9,27,28,29,30,31], 230 patients with multiple sclerosis [16,32,33,34,35], and 332 patients with PD [13,18,36,37,38]. The substantial predominance of patients with neurological conditions may indicate growing interest in the use of the iTUG test to assess mobility in this group.

In most of the analyzed articles, iTUG assessments were conducted in accordance with the recommendations of Podsiadlo [8]. However, in some studies, modifications to the protocol were introduced, such as changes in walking distance or instructions regarding movement speed. In four articles, the walking distance was increased from 3 m to 7 m [13,25,34,38], and in one article to 6 m [18], which may be advantageous due to the recording of a greater number of step cycles. This enables a more precise analysis of gait while making direct comparisons between studies more difficult. Importantly, differences in walking distance do not only increase the amount of collected data but may also systematically influence the relative contribution of individual test phases. Longer walking distances may reduce the proportional impact of transitional phases (e.g., sit-to-stand), while amplifying steady-state gait characteristics, which may lead to inconsistencies when comparing studies using different protocols.

Another important methodological difference between studies was the use of different measurement systems, consisting of varying numbers of IMU sensors and their differing placement. The systems used included G-Walk [11,26,27,28,29,30,31,32,33,37], Mobility Lab [9,13,18,24,25,34,35,36,38], and Noraxon [16,23].

It is worth noting that these systems differ substantially in terms of the number of sensors used and the scope of recorded data. For example, the G-Walk system is based on a single sensor placed in the pelvic region, whereas systems such as Noraxon allow the use of up to between 10 and 20 IMU sensors, enabling a more detailed analysis of movement kinematics. These differences may affect the range and accuracy of the derived parameters, thereby complicating direct comparisons between studies. In addition to hardware-related differences, variations in data processing approaches and proprietary algorithms used for signal segmentation and phase detection may further contribute to inconsistencies between studies.

Despite the wide range of analyzed parameters, a lack of consistency in their reporting across studies can be observed. Many parameters were analyzed only in selected populations or in single studies, which substantially limits the possibility of direct comparison. Importantly, the reported iTUG parameters differ in their sensitivity to methodological heterogeneity. Temporal parameters, such as total test duration and phase durations, appear relatively robust across different sensor configurations and test protocols, whereas spatiotemporal and turning-related parameters are more strongly influenced by methodological factors.

Based on the synthesis of the included studies, iTUG parameters can be broadly categorized according to their sensitivity to methodological variability. Temporal parameters demonstrate relatively high robustness, as they are consistently reported and less dependent on sensor configuration or algorithmic processing. In contrast, spatiotemporal gait parameters (e.g., cadence, gait speed) and turning-related parameters (e.g., angular velocity, peak turning velocity) appear to be more susceptible to methodological factors, including sensor placement, sampling frequency, and signal processing techniques.

This distinction is critical for interpreting the literature, as parameters that are highly protocol-dependent may reflect methodological artifacts rather than true differences between populations. Consequently, caution is required when comparing such parameters across studies or when attempting to establish normative ranges.

In many of the analyzed studies, attention was focused on parameters related to the duration of each iTUG phase [9,11,13,16,23,24,27,29,30,31,32,33,34,35,36,37,38]. The most frequently analyzed parameters include total test duration [9,11,16,23,24,27,28,29,30,31,32,33,35], as well as durations of sit-to-stand [9,11,16,23,24,27,28,29,30,31,32,33,35,36,37,38], turning 1 [11,16,23,24,27,28,30,31,32,33,35], and turning 2 [11,16,23,27,28,30,31,32,33,35,38]. In contrast, among spatiotemporal parameters, cadence was the most frequently analyzed parameter [13,16,23,24,25,27,34,36,37]. Spatiotemporal parameters of gait such as step length [25,36,37] or walking cycle time [34,37], were reported less frequently and less consistently across studies. In turn, among turning-related parameters, the most frequently reported parameter was maximum turning velocity during turning 2 [9,18,23,28,33,37,38].

The predominance of temporal parameters may be related to their relatively simple derivation and high interpretability.

Interest in turning-related parameters may result from their high sensitivity in assessing mobility impairments [12,16,54]. The turning phase consists of several components that require coordination, balance, and postural control. For this reason, this phase is considered more susceptible to impairments and is regarded as a reliable indicator of dynamic balance [12]. At the same time, the observed variability in the reporting of turning-related parameters, such as mean velocity, maximum velocity, or their averaged values, further indicates a lack of standardization in iTUG analysis.

From a clinical perspective, not all of the iTUG parameters that are frequently reported provide the same level of diagnostic information. While many parameters are described in the literature, only a subset appears to have consistent clinical relevance across different populations.

Temporal parameters, particularly total test duration and phase durations, appear to be the most clinically robust and practical indicators of overall mobility impairment [11,16,31,35]. Their widespread use, ease of interpretation make them suitable for routine clinical application.

In contrast, turning-related parameters-especially peak or maximum turning velocity-emerge as particularly sensitive indicators of dynamic balance [12]. These parameters appear to be especially valuable in neurological populations, such as patients with Parkinson’s disease [18,38] or multiple sclerosis [16,35], where impairments in turning are often among the earliest and most pronounced functional deficits.

On the other hand, spatiotemporal gait parameters derived from iTUG, such as stride length [36], gait cycle time [34], or even cadence [16,27,34], although potentially informative, appear to have more limited clinical applicability within the iTUG framework. This is primarily due to their lower reporting consistency, higher sensitivity to methodological variability, and the fact that they can be more accurately assessed using dedicated gait analysis protocols. In addition, it should be noted that in the TUG test, gait is typically analyzed over a 3 m distance, which may be too short a distance to assess gait parameters at the preferred walking speed.

In summary, the results of this review indicate the clinical utility of the individual parameters of the iTUG test. The most important components are the overall time indices, followed by the time parameters for individual phases and the indices related to the performance of turns.

The extensive tabular summaries presented in this review provide a comprehensive overview of reported parameter values; however, they should be interpreted with caution. Due to the methodological heterogeneity described above, these tables represent an aggregation of heterogeneous datasets rather than directly comparable measurements.

Therefore, the reported parameter ranges should not be interpreted as normative reference values, but rather as indicative of the variability present in the current literature. Part of the observed variability may reflect methodological differences rather than true clinical variability.

These observations are supported by findings from studies involving various clinical populations. Significant differences in turning duration were observed in patients with multiple sclerosis in studies by Szaflik et al. [16] and Hershkovitz et al. [35]. Similar results were also obtained in patients with PD, where Dewey et al. [18] and Robertson-Dick et al. [38] reported significant differences in turning duration compared with the control group. Similar observations regarding turning parameters were also reported by Mulas et al. [27] in studies involving older adults.

For parameters such as total test duration in patients with multiple sclerosis, Hershkovitz et al. [35] reported significant differences between patients and healthy individuals. However, Szaflik et al. [16] and Pau et al. [32] did not confirm these differences.

A similar inconsistency in results in patients with multiple sclerosis was observed for the sit-to-stand phase, with significant differences reported by Szaflik et al. [16], whereas Pau [32] and Hershkovitz et al. [35] did not observe such differences. For the stand-to-sit phase, none of the authors reported significant differences [16,32,35]. Similarly, for PD, no significant differences were found for the sit-to-stand and stand-to-sit phases. These observations may suggest that turning-related parameters may represent more sensitive and reliable indicators of mobility impairments than traditional total durations or sit-to-stand and stand-to-sit phases, particularly in clinical populations with varying levels of dysfunction.

The frequency of analysis of individual parameters presented in Table 7 enables the identification of the parameters most commonly used in studies involving specific clinical populations. In patients with PD, the most frequently analyzed parameters included total test duration [13,36,37,38], sit-to-stand duration [36,37,38], cadence [13,36,37], double support [13,36,37] and turning 2 peak/max velocity [18,37,38]. In studies involving multiple sclerosis, the most frequently analyzed parameter is total test duration [11,16,32,33,34,35] along with subphase durations, specifically durations of sit-to-stand [11,16,32,33,35], turning 1 [11,16,32,33,35], turning 2 [11,16,32,33,35] and stand-to-sit phases [11,16,32,35]. In older adults, the most frequently analyzed parameters included total test duration [9,27,29,30,31], sit-to-stand duration [9,27,28,29,30,31], turning 1 [27,28,30,31] and turning 2 durations [27,28,30,31] and the duration of stand-to-sit phase [27,28,30,31]. In turn, in studies involving healthy adults, analyses involved cadence [23,24,25], total test time [23,24], sit-to-stand [23,24], turning 1 [23,24] and stand-to-sit [23,24] durations.

### 4.1. Limitations and Directions of Further Research

A limitation of this literature review is the lack of methodological uniformity, including the use of different equipment and sensor placements, as well as variability in iTUG protocol across studies. The review involved different walking speeds and distances. There is considerable variability in the reported parameters across studies, indicating a lack of standardization in iTUG analysis and limiting the possibility of establishing reference values across different clinical populations.

Moreover, the heterogeneity of measurement protocols may have influenced not only the variability of reported values but also the detectability of between-group differences, which should be taken into account when interpreting the findings of this review. Another limitation of the study was the exclusive use of IMU sensors, without the use of measurement mats or other systems.

A quantitative meta-analysis was not performed due to substantial heterogeneity in study populations, protocols, sensor configurations, and outcome definitions. In addition, many parameters were reported in a limited number of studies or were not consistently defined, which precluded meaningful pooling of results.

Future studies should aim to develop recommendations for a standardized iTUG protocol including a uniform distance and set of analyzed parameters.

In addition, future research should aim to establish consensus guidelines for iTUG assessment, including standardized protocols and reporting, to improve reproducibility and facilitate cross-study comparisons.

Finally, the possibility of publication bias cannot be excluded, as studies reporting statistically significant or clinically relevant findings are more likely to be published, which may have influenced the overall conclusions of this review.

### 4.2. Clinical Implications and Practical Applications

The compiled set of parameters for different conditions at various stages may assist physicians in interpreting iTUG results and in identifying movement impairments characteristic of specific disease entities.

The use of iTUG enables the identification of subtle movement impairments that may not be detectable in standard clinical assessment. The iTUG parameters can be used not only to assess functional status, but also to monitor disease progression and the effects of therapeutic interventions.

The results indicate a need to standardize the iTUG protocol, particularly with regard to participant instructions, number of trials, walking distance, and the analyzed test phases. Standardization of measurement procedures could improve the comparability of results across studies and enhance their clinical utility.

## 5. Conclusions

The analysis revealed substantial methodological heterogeneity across studies, including differences in sensor placement, walking distance, and reported parameters, which limits the possibility of direct comparison of results. The observed variability indicates the dynamic development of the iTUG test and its adaptation to new measurement technologies.

Turning-related parameters in the iTUG test demonstrate a greater ability to detect mobility impairments than traditional temporal measures, such as total test duration or the sit-to-stand and stand-to-sit phases. The differences observed in results for populations with multiple sclerosis, PD, and older adults highlight the importance of the turning phase as a reliable component of motor function assessment.

The results indicate that the analysis of iTUG parameters may serve as a valuable tool to support mobility assessment, as well as the monitoring of functional status and progression of movement impairments across different patient populations.

## Figures and Tables

**Table 1:** Inclusion and exclusion criteria for articles included in the review.

PICOS Category	Inclusion Criteria	Exclusion Criteria
P (Population)	Adults (>18 years): healthy individuals, older adults, and patients with neurological or geriatric conditions	Children and teenagers; animal populations; in vitro studies
I (Intervention)	The Instrumented Timed Up and Go (iTUG) or the TUG test performed using commercially available systems based on inertial measurement units (IMUs)	Classic TUG without instrumentation, other physical therapy tests, commercially available systems based on inertial measurement units (IMU)
C (Comparators)	Healthy vs. clinical groups, different stages of the disease, different age groups, comparison with reference values or the standard TUG time	A small research group (e.g., *n* = 1)
O (Outcomes)	Parameters from the walking and turning phases obtained from the Instrumented Timed Up and Go (iTUG) test, calculated based on data from a commercial IMU system.	Parameters calculated exclusively using proprietary algorithms or previously developed algorithms available in the literature, without the use of a commercial detection system, articles reporting only raw signals
S (Study designs)	Cross-sectional studies, research using normative data	Case reports, review articles, conference abstracts, methodological studies, cohort studies

**Table 2:** Quality assessment scores for the included studies.

Authors	Q1	Q2	Q3	Q4	Q5	Q6	Q7	Q8	How Many Times Will Yes Perform?
Kowal et al. (2025) [23]	Yes	Yes	Yes	Yes	No	No	Yes	Yes	6
Coulthard et al. (2015) [24]	Yes	Yes	Yes	No	No	No	Yes	Yes	5
Abualait et al. (2021) [25]	Yes	Yes	Yes	No	No	No	Yes	Yes	5
Santos et al. (2025) [26]	Yes	Yes	Yes	Unclear	No	No	Yes	Yes	5
Mulas et al. (2020) [27]	Yes	Yes	Yes	Yes	No	No	Yes	Yes	6
Zarzeczny et al. (2017) [28]	Yes	Yes	Yes	No	No	No	Yes	Yes	5
Park et al. (2025) [29]	Yes	Yes	Unclear	Yes	No	No	Yes	Yes	5
Porta et al. (2018) [30]	Yes	Yes	Yes	Yes	Yes	No	Yes	Yes	7
Pau et al. (2022) [31]	Yes	Yes	Yes	Yes	No	No	Yes	Yes	6
Seo et al. (2019) [9]	Unclear	Yes	Yes	Unclear	No	No	Yes	Yes	4
Pau et al.(2020) [32]	Yes	Yes	Yes	Yes	Yes	Yes	Yes	Yes	8
Lorefice et al. (2017) [33]	Yes	Yes	Yes	Yes	No	No	Yes	Yes	6
Craig et al. (2017) [34]	Yes	Yes	Yes	No	No	No	Yes	Yes	5
Pau et al. (2017) [11]	Yes	Yes	Yes	Yes	No	No	Yes	Yes	6
Hershkovitz et al. (2019) [35]	Yes	Yes	Yes	Unclear	No	No	Yes	Yes	5
Szaflik et al. (2026) [16]	Yes	Yes	Yes	Yes	No	No	Yes	Yes	6
Cakmak et al. (2023) [36]	No	Yes	Yes	No	No	No	Yes	Yes	4
Atterbury et al. (2017) [13]	Yes	Yes	Yes	No	No	No	Yes	Yes	5
Dewey et al. (2014) [18]	Yes	Yes	Yes	Unclear	No	No	Yes	Yes	5
Mostile et al. (2023) [37]	Yes	Yes	Yes	Yes	Yes	Yes	Yes	Yes	8
Robertson-Dick et al. (2023) [38]	Yes	Yes	Yes	No	No	No	Yes	Yes	5
Huisinga et al. (2018) [39]	Yes	Yes	Yes	No	No	No	Yes	Yes	5
Celletti et al. (2021) [40]	Yes	Yes	No	Yes	Yes	No	Yes	Yes	6
Spina et al. (2022) [41]	Yes	Yes	Yes	Yes	No	No	Yes	Yes	6
Kowal et al. (2023) [42]	Yes	Yes	Yes	Yes	No	No	Yes	Yes	6
Monaghan et al. (2024) [43]	Yes	Yes	Yes	Yes	No	No	No	Yes	5
Cimolin et al. (2019) [44]	Yes	Yes	Yes	Yes	No	No	Yes	Yes	6
Tajuddin et al. (2021) [45]	Yes	Yes	Yes	Yes	No	No	Yes	Yes	6
Arippa et al. (2024) [46]	Yes	Yes	Yes	Unclear	No	No	Yes	Yes	5
O’Keefe et al. (2016) [47]	Yes	Yes	Yes	No	No	No	Yes	Yes	5
Celletti et al. (2020) [14]	Yes	Yes	Yes	Yes	No	No	Yes	Yes	6
Familiari et al. (2024) [48]	Yes	Yes	Yes	Yes	No	No	Yes	Yes	6
Buraschi et al. (2022) [49]	Yes	Yes	Yes	Yes	No	No	Yes	Yes	6
Mostile et al. (2021) [50]	Yes	Yes	Yes	No	Yes	No	Yes	Yes	6
Brognara et al. (2022) [51]	Yes	Yes	Yes	No	No	No	Yes	Yes	5
Statland et al. (2019) [52]	Yes	Yes	Yes	No	No	No	Yes	Yes	5

**Table 3:** iTUG measurement methodology and study group characteristics (* Study also included patients with fragile X-associated tremor/ataxia syndrome (FXTAS); results for FXTAS are described in Section 3.6), (HC—healthy control group, PD—Parkinson’s disease, MS—multiple sclerosis, F—female, M—male, EDSS—Expanded Disability Status Scale, N/A—this is not a number, no information available).

Authors	Clinical Population	Group of Healthy Individuals/Control Group	System and Location	iTUG Test Procedure
Kowal et al. (2025) [23]	N/A	73 participants (37 women and 36 men), 31 ± 5.5 years, 68.4 ± 4.3 kg, 176.2 ± 9.1 cm	Noraxon (Scottsdale, AZ, USA), which included 18 wireless IMUs capable of capturing three-dimensional motion data at a sampling rate of 200 Hz	self-selected normal walking speed, walks 3 m
Coulthard et al. (2015) [24]	N/A	20 young adult participants (10 male)—mean age: 22.5 ± 2.6 years,	using the Mobility Lab iTUG sensors (OPAL, APDM Inc., Portalnd, OR, USA). Each participant wore a total of six sensors positioned on the posterior side of both wrists, directly on top of the distal sternum, posteriorly on the lower lumbar vertebra, and anteriorly on each ankle just proximal to the malleoli. To export data use Mobility Lab system	walk at their normal, comfortable pace for all trials, 3.5 m Different trials: Control and mental tracking (MT) task which involved counting backwards by seven’s out loud from a given three-digit number.
Abualait et al. (2021) [25]	N/A	20 healthy subjects (14 women and 6 men; with a mean and standard deviation (SD) age of 22.8 (4.42) years)	Mobility Lab System (APDM Inc., Portland, OR, USA), one sensor was placed on the sternum 2 cm below the sternal notch and the last sensor was placed at the midline on the lumbar spine at L5	walk 7 m, the modified iTUG under three conditions: (1) without ADs (NAD), (2) with a single-tip cane (CAD); and (3) with a four-wheeled walker (WAD)
Santos et al. (2025) [26]	N/A	Females (n = 53; 67.09%), age: 40.36 (13.35) Males (n = 26; 32.91%), age: 38.72; (12.84)	G-Walk (BTS Bioengineering Corp., Quincy, MA, USA)., belt on the volunteers’ waist, covering the intervertebral space between S1	3 m, self-selected comfortable pace
Mulas et al. (2020) [27]	Cognitively impaired young-old (age ≤ 75, CI-YO): ACE-R score < 79 (n = 28) Cognitively impaired old-old (age > 75, CI-OO): ACE-R score < 60 (n = 43)	Healthy controls young-old (age ≤ 75, HC-YO): ACE-R score ≥ 79 (n = 64) Healthy controls old-old (age > 75, HC-OO): ACE-R score ≥ 60 (n = 78)	G-Walk (G-Sensor, BTS Bioengineering S.p.A., Garbagnate Milanese, Italy)—_location: L1 vertebrae	walked for 3 m at a comfortable and safe speed
Zarzeczny et al. (2017) [28]	twenty-six women, 80 years of age or older—mean 85.8 ± 3.6	N/A	G-Sensor, (BTS Bioengineering S.p.A., Italy), fixed with an elastic belt at the level of lumbar segment L5 over the subject’s clothes	walk as fast as was safely possible to a line on the floor three meters away, make a 180° turn and walk back to the chair and sit down again
Park et al. (2025) [29]	30 participants per group: the tai chi, strength exercise, yoga groups, home exercise. The mean age of the participants was 68.4 ± 7.5 years.	N/A	commercial wearable inertial sensor G-WALK(G-Sensor, BTS Bioengineering S.p.A., Italy)	walked around the cone located 3 m at the typical walking speed
Porta et al. (2018) [30]	125 participants was stratified into four groups as follows: Group 1: <70 years (n = 31; 20 F, 11 M); Group 2: 71–75 years (n = 32; 19 F, 13 M); Group 3: 76–80 years (n = 32; 19 F, 13 M); Group 4: >80 years (n = 30; 14 F, 16 M).	N/A	G-Sensor, BTS Bioengineering S.p.A., Italy, was attached to the subject’s waist using a semi-elastic belt approximately at the L4–L5 intervertebral space position	walked for 3 m at a comfortable and safe speed
Pau et al. (2022) [31]	51 women aged over 65 (mean age 77.9 ± 5.2 years, based on subjective cognitive complaints and slow gait, they were assigned either to The Motoric Cognitive Risk (MCR, n = 24) or non-The Motoric Cognitive Risk (non-MCR, n = 27) group	N/A	wearable inertial sensor (G-Sensor, BTS Bioengineering S.p.A., Italy), L1 vertebrae locations	walked for 3 m at a comfortable and safe speed
Seo et al. (2019) [9]	26 (4 male, 22 female) fallers age: 73.84 ± 5.19 and 43 non-fallers (11 male, 32 female) age: 76.32 ± 4.78	N/A	Opal IMU sensor module (APDM Inc. Portland, OR, USA) and mobility lab system were used,	3 m
Pau et al.(2020) [32]	49 people Multiple Sclerosis, 31 Women (47.8 ± 12.2, EDSS 3.3 ± 1.9) and 18 Man (53.5 ± 11.5, EDSS 3.6 ± 1.3)	N/A	inertial sensor G-Sensor^®^, BTS Bioengineering S.p.A., Italy, the device was attached to the participant’s back approximately at L1 vertebra	walked straight for 3 m at a comfortable and safe speed
Lorefice et al. (2017) [33]	60 MS (19 male), 41.5 ± 11.6 years old and EDSS 2.3 ± 1.2	N/A	(G-Sensor^®^. BTS Bioengineering S.p.A., Italy). The device was laced to the subject using a belt placed at L4–L5, providing acceleration values along three orthogonal axes	walk straight ahead at your normal pace, walk 3 m
Craig et al. (2017) [34]	Fifteen MS between 20 and 60 years old (12 F/3 M, age: 48.2 (8.7))	HC—15 people—12 F/3 M, 47.8 (9.5)	6 wireless inertial sensors (Opal sensors, APDM, Portland, OR, USA)	walk at a normal pace to a point on the floor 7 m in front of them, turn around, walk at a normal pace back to the chair, and sit back down in the chair with minimal use of their hands.
Pau et al. (2017) [11]	Class 1: low-minimal disability (EDSS 0−1.5, n = 57), (39 F, 18 M), age: 39.8 (8.2) Class 2: mild disability (EDSS 2−3.5, n = 32), (22 F, 10 M), age:43.5 (9.5) Class 3: moderate disability (EDSS 4−6.5, n = 17), (12 F, 5 M), age:48.6 (10.1)	HC—42 (16 F, 26 M), age: 39.6 (13.5)	G-Sensor^®^, BTS Bioengineering S.p.A., Italy, the L4–L5 inter-vertebral space	walked straight for 3 m at a comfortable and safe speed
Hershkovitz et al. (2019) [35]	Fifty PwMS (33 women and 17 men), aged 44.2 (S.D = 7.2), were recruited from the Multiple Sclerosis Center, Fallers (n = 16); Median EDSS = 4.5 [2.5, 5.5] Non-fallers (n = 34) Median EDSS = 3.5 [2.0, 5.5]	Twenty-five healthy subjects (18 women and 7 men), aged 44.4 (S.D = 8.6),	APDM Mobility Lab System (v2) (Opal sensors, APDM, Portland, OR, USA).	3 m walk at a normal pace
Szaflik et al. (2026) [16]	30 patients with MS (19 Female/11 Male), Age (mean ± SD):33.83 ± 10.01 years	30 healthy people (18 Female/12 Male), Age (mean ± SD): 30.13 ± 11.74 years	Noraxon MyoMotion MR4.0.90, on the upper spine (C7 circle) and feet	3 m
Cakmak et al. (2023) [36]	20 patients with PD (8 F/12 M), age: 69.10 (6.92) 13 diagnosis of probable NPH (6 F/7 M), age: 71.92 (4.11)	13 Healthy control (6 F/7 M), age: 69.23 (9.03)	APDM Mobility Laboratory System (APDM Inc., Portland, OR, USA), IMU sensors were attached to the participants’ feet and lumbar	3 m
Atterbury et al. (2017) [13]	40 participants with confirmed idiopathic PD, between 50 and 80 years, with Hoehn and Yahr (H&Y) Stages I–III (mild to moderate) PD. Participants were divided into a therapist-supervised group (TS, n = 24, age: 65.0 ± 8.2) and a home group (HB, n = 16, age: 65.0 ± 7.1). The groups participated in an eight-week balance training program with a physical therapist or used a DVD.	N/A	Mobility Lab™, APDM^®^, USA	walk 7 m, at their comfortable walking pace
Dewey et al. (2014) [18]	135 PD patients—age: 64.0 ± 9.9, Hoehn & Yahr = 2.0 ± 0.6, The group was divided into a group with normal gait and a group with normal balance.	Controls n = 66, age: 62.9 ± 9.5	Six movement sensors called Opals, (Mobility Lab, APDM Inc., Portland, OR, USA). one on each ankle and wrist, the lower back, and the upper chest.	walked 6 m
Mostile et al. (2023) * [37]	A total of 42 patients, 21 diagnosed as Idiopathic normal pressure hydrocephalus (iNPH), and 21 diagnosed as PD, iNPH-P (N = 21)—71.42 (10.68), and PD (N = 21)—60.57 (10.01)	N/A	G-WALK, BTS Bioengineering S.p.A, Italy	walk 3 m, in accordance with podsiadło
Robertson-Dick et al. (2023) * [38]	The study included 22 participants with Fragile X-associated tremor/ataxia syndrome (age: 69.14 ± 8.12), 23 with PD (age: 71.26 ± 7.87), 20 with essential tremor (age: 69.80 ± 8.85)	20 controls, age: 62.65 ± 8.52	APDM Mobility Lab™ six inertial sensor system (APDM™; Portland, OR, USA)	self-selected speed, walk at 7 m

**Table 4:** Duration of iTUG and its subphases (* Study also included patients with fragile X-associated tremor/ataxia syndrome (FXTAS); results for FXTAS are described in Section 3.6, CI-YO—Cognitively impaired young-old, CI-OO—Cognitively impaired old-old, HC-YO—Healthy controls young-old, NAD—iTUG without ADs, CAD—iTUG with a single-tip cane, WAD—iTUG with a four-wheeled walker).

Authors	Total Duration [s]		Stand Duration [s]		Walk 1 Duration [s]		Turn 1 Duration [s]		Walk 2 Duration [s]		Walking Time [s]		Turn 2 Duration [s]		Turn Time [s]		Sit Phase Duration [s]	
	Clinical Population	Healthy Controls	Clinical Population	Healthy Controls	Clinical Population	Healthy Controls	Clinical Population	Healthy Controls	Clinical Population	Healthy Controls	Clinical Population	Healthy Controls	Clinical Population	Healthy Controls	Clinical Population	Healthy Controls	Clinical Population	Healthy Controls
Kowal et al. (2025) [23]		13.1 ± 1.9		1.1 ± 0.3		4.1 ± 0.9		2 ± 0.4		3.2 ± 0.7				1.1 ± 0.3				1.6 ± 0.4
Coulthard et al. (2015) [24]		Control: 15.6 ± 1.4 MT: 17.36 ± 1.7		Control: 2.21 ± 0.23 MT: 2.29 ± 0.19				Control: 1.68 ± 0.29 MT: 1.71 ± 0.31				Control: 11.7 ± 1.3 MT: 13.6 ± 1.6						Control: 3.49 ± 0.6 MT: 3.75 ± 0.48
Abualait et al. (2021) [25]																		CAD: 5.09 ± 1.07 WAD: 5.75 ± 1.02 NAD: 3.64 ± 0.44
Mulas et al. (2020) [27]	CI-YO 15 ± 2.9 CI-OO 21.7 ± 8.5	HC-YO—11.1 ± 1.7 HC_OO—12.3 ± 2.0	CI-YO 2.2 ± 1.1 CI-OO 2.2 ± 0.9	HC-YO—1.5 ± 0.3 HC_OO—1.5 ± 0.2			CI-YO 2.7 ± 0.6 CI-OO 3.9 ± 1.4	HC-YO—2.2 ± 0.4 HC_OO—2.5 ± 0.7					CI-YO 2.0 ± 0.6 CI-OO 2.7 ± 1.3	HC-YO—1.7 ± 0.5 HC_OO—2.0 ± 0.7			CI-YO 1.3 ± 0.6 CI-OO 1.4 ± 0.6	HC-YO—0.9 ± 0.3 HC_OO—0.9 ± 0.3
Zarzeczny et al. (2017) [28]			2.18 ± 0.97				4.76 ± 4.16				8.64 ± 6.47		4.99 ± 2.07				2.75 ± 0.98	
Park et al. (2025) [29]	Home exercise: 9.10 ± 1.63 Tai chi: 9.38 ± 1.55 Strength exercise: 9.90 ± 4.52 Yoga: 8.24 ± 1.86		Home exercise: 1.33 ± 0.18 Tai chi: 1.34 ± 0.19 Strength exercise: 1.30 ± 0.28 Yoga: 1.15 ± 0.19		Home exercise: 1.70 ± 0.54 Tai chi: 1.74 ± 0.47 Strength exercise: 2.11 ± 1.20 Yoga: 1.61 ± 0.68				Home exercise: 1.46 ± 0.52 Tai chi: 1.48 ± 0.55 Strength exercise: 1.46 ± 1.04 Yoga: 1.34 ± 0.67									
Porta et al. (2018) [30]	Group 1: <0 years—F: 10.11 ± 2.35 M: 9.74 ± 2.06 Group 2: 71–75 years F: 10.45 ± 2.73 M: 10.61 ± 2.51 Group 3: 76–80 years F: 13.08 ± 2.15, M: 13.38 ± 3.30 Group 4: >80 years F: 16.93 ± 3.38 M: 14.63 ± 3.17		Group 1: <70 years—F: 1.31 ± 0.38 M: 1.38 ± 0.35 Group 2: 71–75 years F: 1.66 ± 0.64 M: 1.51 ± 0.45 Group 3: 76–80 years F: 1.83 ± 0.49, M: 1.64 ± 0.53 Group 4: >80 years F: 2.00 ± 0.67, M: 1.87 ± 0.60				Group 1: <70 years—F: 2.09 ± 0.91 M: 2.44 ± 0.71 Group 2: 71–75 years F: 2.69 ± 1.04 M: 2.73 ± 1.07 Group 3: 76–80 years F: 3.83 ± 1.01 M: 3.50 ± 1.51 Group 4: >80 years F: 4.59 ± 1.35 M: 4.31 ± 0.94						Group 1: <70 years—F: 1.98 ± 1.33 M: 2.35 ± 0.73 Group 2: 71–75 years F: 2.65 ± 1.40 M: 2.24 ± 0.87 Group 3: 76–80 years F: 3.82 ± 0.87 M: 3.40 ± 1.20 Group 4: >80 years F: 4.76 ± 1.11 M: 3.87 ± 1.04				Group 1: <70 years—F: 1.61 ± 0.49 M: 1.85 ± 0.53 Group 2: 71–75 years F: 1.76 ± 0.50 M: 1.89 ± 1.10 Group 3: 76–80 years F: 2.30 ± 0.61 M: 2.33 ± 0.70 Group 4: >80 years F: 2.43 ± 0.73 M: 2.58 ± 0.79	
Pau et al. (2022) [31]	non-MCR: 14.15 ± 2.61 MCR: 22.39 ± 7.55		non-MCR: 1.81 ± 0.57 MCR: 2.63 ± 1.15				non-MCR: 2.68 ± 0.89 MCR: 3.84 ± 1.49						non-MCR: 2.06 ± 0.79 MCR: 2.9 ± 1.09				non-MCR: 1.08 ± 0.36 MCR: 1.55 ± 0.86	
Seo et al. (2019) [9]	Faller: 13.25 ± 1.46 Non-faller: 13.20 ± 1.30		Faller: 2.19 ± 0.32 Non-faller: 2.28 ± 0.49															
Pau et al. (2020) [32]	Women 13.92 ± 6.5 Men 12.68 ± 3.83		Women 1.64 ± 0.64 Men 1.49 ± 0.42				Women 2.72 ± 2.3 Men 2.81 ± 1.73				Women 6.62 ± 3.65 Men 5.33 ± 1.72		Women 1.91 ± 0.99 Men 2.10 ± 0.72				Women 1.03 ± 0.45 Men 0.97 ± 0.21	
Lorefice et al. (2017) [33]	14.02 ± 7.08		1.67 ± 0.51				2.21 ± 0.8						2.03 ± 0.95					
Craig et al. (2017) [34]	17.93 ± 2.31	17.05 ± 2.52																
Pau et al. (2017) [11]	Class 1: 12.9 ± 2.3 Class 2: 16.5 ± 5.0 Class 3: 18.2 ± 5.8	12.6 ± 1.8	Class 1: 1.8 ± 0.4 Class 2: 1.8 ± 0.4 Class 3: 2.1 ± 0.4	1.8 ± 0.5			Class 1: 2.8 ± 0.5 Class 2: 3.3 ± 1.0 Class 3: 3.3 ± 0.6	2.6 ± 0.5					Class 1: 3.0 ± 0.5 Class 2: 3.2 ± 0.8 Class 3: 3.6 ± 0.7	2.8 ± 0.5			Class 1: 2.0 ± 0.5 Class 2: 2.2 ± 0.7 Class 3: 2.3 ± 0.7	2.1 ± 0.6
Hershkovitz et al. (2019) [35]	10.40 ± 2.0 Fallers 11.4 ± 1.7 Non-fallers 10.0 ± 2.0	7.5 ± 0.6	0.96 ± 0.15 Fallers 1.00 ± 0.17 Non-fallers 0.95 ± 0.14	0.85 ± 0.12			2.49 ± 0.54 Fallers 2.53 ± 0.41 Non-fallers 2.42 ± 0.52	2.25 ± 0.34					1.80 ± 0.41 Fallers 1.87 ± 0.41 Non-fallers 1.77 ± 0.42	1.46 ± 0.16			0.72 ± 0.13 Fallers 0.72 ± 0.13 Non-fallers 0.72 ± 0.13	1.5 ± 0.3
Szaflik et al. (2026) [16]	8.78 ± 1.2	8.7 ± 1.18	1.38 ± 0.17	1.21 ± 0.22			1.62 ± 0.18	1.9 ± 0.29			3.43 ± 0.93	2.99 ± 0.85	0.89 ± 0.29	1.13 ± 0.16			1.46 ± 0.23	1.47 ± 0.3
Cakmak et al. (2023) [36]	PD 10.81 ± 2.92 NPH 14.78 ± 4.04	11.67 ± 2.85	PD 1.17 ± 0.39 NPH 1.12 ± 0.1	0.99 ± 0.12											PD 2.31 ± 0.54 NPH 2.77 ± 0.54	2.46 ± 0.6	PD 0.92 ± 0.25 NPH 0.99 ± 0.26	0.87 ± 0.13
Atterbury et al. (2017) [13]	TS pre 19.00 ± 3.01 TS post 19.14 ± 3.29 HB pre 22.96 ± 10.04 HB post 22.89 ± 0.58																	
Dewey et al. (2014) [18]															PD: 2.65 ± 0.72 PD normal gait: 2.36 ± 0.57 PD normal balance: 2.58 ± 0.61	2.27 ± 0.59		
Mostile et al. (2023) * [37]	PD—1.93 ± 0.49		PD—1.93 ± 0.49															
Robertson-Dick et al. (2023) * [38]	PD: 20.02 ± 2.64	17.71 ± 2.28	PD: 2.49 ± 0.23	2.28 ± 0.30									PD: 4.56 ± 0.98	4.21 ± 0.65				

**Table 5:** Spatiotemporal parameters obtained during the walking phase of the iTUG (* Study also included patients with fragile X-associated tremor/ataxia syndrome (FXTAS); results for FXTAS are described in Section 3.6, CI-YO—Cognitively impaired young-old, CI-OO—Cognitively impaired old-old, HC-YO—Healthy controls young-old, NAD—iTUG without ADs, CAD—iTUG with a single-tip cane, WAD—iTUG with a four-wheeled walker).

Authors	Mean Walking Speed [m/s]		Walking Cycle Time [s]		Stride Length [m]		Cadence [Steps/min]		Stance Time [%]		Swing Time [%]		Double Support [%]	
	Clinical Population	Healthy Controls	Patients	Healthy Controls	Patients	Healthy Controls	Patients	Healthy Controls	Patients	Healthy Controls	Patients	Healthy Controls	Patients	Healthy Controls
Kowal et al. (2025) [23]								107.6 ± 8.3		L: 60.3 ± 2.0 R: 59.6 ± 2.3		L: 39.7 ± 2.0 R: 40.5 ± 2.3		
Coulthard et al. (2015) [24]								Control: 112 ± 5.4 MT: 105.9 ± 7.4		Control: 60.8 ± 1.8 MT: 61.8 ±2.1				Control: 21.6 ± 3.6 MT: 23.7 ± 4.2
Abualait et al. (2021) [25]		CAD: 0.56 ± 0.21 WAD: 1.01 ± 0.16 NAD: 1.26 ± 0.09				CAD: 1.1 ± 0.17 WAD: 1.27 ± 0.14 NAD: 1.4 ± 0.09		CAD: 59.66 ± 11.58 WAD: 95.19 ± 11.41 NAD: 105.71 ± 9.25						
Mulas et al. (2020) [27]	CI-YO 0.79 ± 0.25 CI-OO 0.60 ± 0.28	HC-YO 1.10 ± 0.14 HC_OO 0.95 ± 0.21					CI-YO 106.58 ± 13.31 CI-OO 98.88 ± 12.32	HC-YO—114.42 ± 8.28 HC_OO—109.87 ± 6.6					CI-YO 22.24 ± 1.73 CI-OO 23.92 ± 2.10	HC-YO 20.42 ± 1.85 HC_OO 21.28 ± 2.00
Craig et al. (2017) [34]			1.02 ± 0.07	1.05 ± 0.07			118.6 ± 8.53	114.95 ± 6.69	60.97 ± 2.3	61.57 ± 1.95	39.03 ± 2.3	38.43 ± 1.95	21.93 ± 4.63	23.13 ± 3.91
Szaflik et al. (2026) [16]							132.01 ± 13.24	117.44 ± 12.77						
Cakmak et al. (2023) [36]	PD 0.980 ± 0.21 NPH 0.620 ± 0.22	0.922 ± 0.20			PD 1.01 (0.940–1.14) NPH 0.771 (0.472–0.975)	1.09 (0.957–1.19)	PD 103.3 ± 13.27 NPH 98.86 ± 16.17	104.1 ± 6.95	PD 61.18 (59.62–62.90) NPH 64.24 (62.99–67.10)	61.02 (60–61.83)	PD 38.82 (37.10–40.38) NPH 35.76 (32.83–37.01)	38.99 (37.99–39.84)	PD 22.32 (19.20–25.95) NPH 28.58 (26–34.37)	22.04 (20–23.60)
Atterbury et al. (2017) [13]							TS pre 112 ± 9.77 TS post 114.82 ± 10.15 HB pre 110.27 ± 14.31 HB post 108.61 ± 8.22						TS pre 23 ± 6.23 TS post 21.47 ± 4.93 HB pre 23.56 ± 8.34 HB post 22.96 ± 5.42	
Mostile et al. (2023) * [37]			PD—0.94 ± 0.28		PD—1.26 ± 0.37		PD—91.47 ± 22						PD—12.15 ± 5.2	

**Table 6:** Turning parameters in the iTUG test (* Study also included patients with fragile X-associated tremor/ataxia syndrome (FXTAS); results for FXTAS are described in Section 3, (1) NAD—iTUG without ADs, (2) CAD—iTUG with a single-tip cane, WAD—iTUG with a four-wheeled walker.

Authors	Turn 1 Average Velocity (°/s)		Turn 1 Peak/Max Velocity (°/s)		Turn 2 Average Velocity (°/s)		Turn 2 Peak/Max Velocity (°/s)		Average Turning Velocity (°/s)	
	Patients	Healthy Controls	Patients	Healthy Controls	Patients	Healthy Controls	Patients	Healthy Controls	Patients	Healthy Controls
Kowal et al. (2025) [23]				67 ± 22.8				67 ± 22.8		
Coulthard et al. (2015) [24]										Control: 206.7 ± 37.4 MT: 209.5 ± 44.8
Abualait et al. (2021) [25]										CAD: 107.88 ± 18.44 WAD: 60.46 ± 15.46 NAD: 147.73 ± 27.35
Santos et al. (2025) [26]		60.0–73.0				72.0–87.0				
Zarzeczny et al. (2017) [28]	36.20 ± 17.06		101.80 ± 40.66		34.13 ± 12.89		92.87 ± 27.31			
Seo et al. (2019) [9]							Faller: 143.82 ± 28.75 Non-faller: 155.45 ± 35.52			
Lorefice et al. (2017) [33]	76.72 ± 27.12		174.67 ± 50.84		82.24 ± 34.86		191.51 ± 54.5			
Hershkovitz et al. (2019) [35]	197.6 ± 50.8 Fallers 185.2 ± 50.8 Non-fallers 201.4 ± 52.5	220.6 ± 40.3			248.0 ± 52.9 Fallers 237.5 ± 56.7 Non-fallers 252.6 ± 51.4	294.6 ± 46.8				
Szaflik et al. (2026) [16]			97.14 ± 36.11	70.78 ± 39.56						
Cakmak et al. (2023) [36]									PD 166.1 ± 40.21 NPH 128.1 ± 43.51	161 ± 43.11
Dewey et al. (2014) [18]							PD: 150.86 ± 38.50 PD normal gait: 167.49 ± 43.77 PD normal balance: 153.79 ± 37.59	178.19 ± 36.40		
Mostile et al. (2023) * [37]	iNPH-P—44.51 ± 11.42, PD—59.05 ± 13.06		iNPH-P—121 ± 35.04, PD—159.65 ± 56.75		iNPH-P—43.77 ± 13.89, PD—67.55 ± 18.91		iNPH-P—112.54 ± 36.62, PD—164.52 ± 52.61			
Robertson-Dick et al. (2023) * [38]							PD: 135.15 ± 36.34	173.98 ± 39.34		

**Table 7:** List of articles reporting analyzed iTUG parameters by study groups (populations).

Parameter	Healthy	Elders	MS	PD
**Time parameters**				
Total duration [s]	[23,24]	[9,27,29,30,31]	[11,16,32,33,34,35]	[13,36,37,38]
Stand duration [s]	[23,24]	[9,27,28,29,30,31]	[11,16,32,33,35]	[36,37,38]
Walk 1 duration [s]	[23]	[29]	Not raported	Not raported
Turn 1 duration [s]	[23,24]	[27,28,30,31]	[11,16,32,33,35]	Not raported
Walk 2 duration [s]	[23]	[29]	Not raported	Not raported
Walking time [s]	[24]	[28]	[16,32]	Not raported
Turn 2 duration [s]	[23]	[27,28,30,31]	[11,16,32,33,35]	[38]
Turn time [s]	Not raported	Not raported	Not raported	[18,36]
Sit phase duration [s]	[23,24]	[27,28,30,31]	[11,16,32,35]	[36]
**Spatiotemporal from the gait**				
Mean walking Speed [m/s]	[25]	[27]	Not raported	[36]
Walking cycle time [s]	Not raported	Not raported	[34]	[37]
Stride length [m]	[25]	Not raported	Not raported	[36,37]
Cadence [steps/min]	[23,24,25]	[27]	[16,34]	[13,36,37]
Stance time [%]	[23,24]	Not raported	[34]	[36]
Swing time [%]	[23]	Not reported	[34]	[36]
Double support [%]	[24]	[27]	[34]	[13,36,37]
**Rotational speed from rotation phase 1 or 2**				
Turn 1 Average Velocity [°/s]	[26]	[28]	[33,35]	[37]
Turn 1 Peak/Max Velocity [°/s]	[23]	[28]	[16,33]	[37]
Turn 2 Average Velocity [°/s]	[26]	[28]	[33,35]	[37]
Turn 2 Peak/Max Velocity [°/s]	[23]	[9,28]	[33]	[18,37,38]
Average Turning Velocity [°/s]	[24,25]	Not reported	Not reported	[36]

## Data Availability

The original contributions presented in this study are included in the article. Further inquiries can be directed to the corresponding author.

## Supplementary Materials

The following supporting information can be downloaded at: https://www.mdpi.com/article/10.3390/jcm15093307/s1, Supplementary Files: PRISMA 2020 Checklist; PRISMA 2020 Abstract Checklist [55].

## Abbreviations

The following abbreviations are used in this manuscript:

IMU	Inertial measurement units
TUG	Timed Up and Go
iTUG	Instrumented Timed Up and Go
MS	Multiple sclerosis
PD	Parkinson’s disease
